# Kikuchi-Fujimoto's Disease or Histiocytic Necrotizing Lymphadenitis Following mRNA COVID-19 Vaccination: A Rare Case

**DOI:** 10.7759/cureus.24155

**Published:** 2022-04-15

**Authors:** Sanae Daghri, Nadia Belmoufid, Amal Rami, Abderahmane Al Bouzidi, Nouama Bouanani

**Affiliations:** 1 Department of Hematology, Faculty of Medicine, Mohammed VI University of Health Sciences (UM6SS), Casablanca, MAR; 2 Department of Radiology, Faculty of Medicine, Mohammed VI University of Health Sciences (UM6SS), Casablanca, MAR; 3 Department of Pathology, Faculty of Medicine, Mohammed VI University of Health Sciences (UM6SS), Casablanca, MAR

**Keywords:** covid-19, coronavirus disease 2019, mrna-based vaccine, cervical lymphadenopathy, histologic diagnosis, histiocytic necrotizing lymphadenitis, s: kikuchi-fujimoto disease

## Abstract

Kikuchi-Fujimoto disease (KFD), also known as necrotizing histiocytic lymphadenopathy, is a rare and benign lymph node disorder that mainly occurs in young women. It is clinically characterized by fever with tender and painful cervical lymphadenopathy mostly; however, all areas of lymph nodes can be involved. This disorder is often mistaken for malignant lymphoma or infection. The precise pathophysiology of KFD remains unknown, but it is theorized that it may be post-viral or associated with an autoimmune disease. The diagnosis is based on the histological analysis of the excised involved lymph node. The treatment is mainly supportive with favorable outcomes within a few weeks or months.

In this case, we present a 24-year-old woman without a past medical history, who consulted for painful bilateral cervical lymphadenopathy associated with fever that has been evolving for one month following the coronavirus disease 2019 (COVID-19) vaccination. The initial diagnostic workup was performed and the diagnosis of KFD was confirmed based on the histopathological findings of the excised lymphadenopathy. Therapeutic management was based on oral corticosteroid treatment with clinical and radiological improvement after a few days without recurrence during follow-up.

This article aims to report a rare case of KFD in a patient after receiving the messenger ribonucleic acid (mRNA)-based COVID-19 vaccine. Therefore, this case highlights the possible association between COVID-19 vaccination and KFD and this should be considered in the differential diagnosis.

## Introduction

Kikuchi-Fujimoto disease (KFD), also known as necrotizing histiocytic lymphadenopathy, is a rare and benign lymph nodes disorder occurring with a large preponderance in young women [1]. KFD is characterized by painful lymphadenopathy, fever, fatigue, and leukopenia [1]. This disorder is commonly misdiagnosed as a viral infection due to the self-limiting evolution of the disease. Clinicians and pathologists are poorly familiar with this entity, which frequently causes significant diagnostic challenges. The etiopathogenesis of KFD remains unclear; an autoimmune response and diverse infectious agents are the most common hypotheses [1,2]. More recently in the coronavirus disease 2019 (COVID-19) pandemic, few cases of KFD after infection have been reported [3,4]. However, to our knowledge, only four cases of KFD following vaccination against COVID-19 have been described to date [5-7].

We present a rare case of a 24-year-old woman with KFD following administration of messenger ribonucleic acid (mRNA) vaccine against COVID-19 to contribute to the literature and raise awareness of this entity in the differential diagnosis of persistent lymphadenopathy.

## Case presentation

A 24-year-old woman without a past medical history, especially no history of severe acute respiratory syndrome coronavirus 2 (SARS-CoV-2) infection presented multiple painful bilateral cervical lymphadenopathy associated with cycles of fever without weight loss or night sweats. She reports receiving the first dose of the mRNA COVID-19 vaccine one month before the start of her symptoms. During the month, there was no change or variation in the size of the enlarged lymph node. Her condition had been previously considered “lymphadenitis” by a primary care physician, without any clinical improvement or resolution of the cervical lymphadenopathy after treatment with an antibiotic course.

On physical examination, she was found to be hemodynamically stable and febrile at 39°C. The patient's neck palpation revealed bilateral cervical lymphadenopathy and was tender to palpation, painful, and non-adherent to deep tissues, with a maximum size of 1.5 cm. There was no evidence of edema, erythema, or warmth. Her nasopharyngeal swab for SARS-CoV-2 polymerase chain reaction (PCR) was negative; SARS CoV-2 IgG antibodies were positive (173 units/ml).

Initial laboratory studies revealed hemoglobin (Hb): 10.9 g/dL, white blood cells (WBC): 2150/mm3 (neutrophils: 1100 /mm3, lymphocytes: 700 /mm3, monocytes: 350 /mm3), platelets: 162 000 /mm3, creatinine: 86 μmol/L, C-reactive protein (CRP): 56 mg/L, lactate dehydrogenase (LDH): 210 units/L (N<250UI/L ), ferritin: 560 ng/dL. Liver blood tests and thyroid function were normal. Peripheral blood did not show any blast or atypical lymphocytes. Blood cultures were negative. Further analysis did not reveal ongoing infections of human immunodeficiency virus (HIV), hepatitis panel, Epstein-Barr virus (EBV), syphilis, and cytomegalovirus (CMV). Quantiferon tuberculosis blood test was negative. Antinuclear antibodies (ANA) and anti-DNA were negative. 

**Figure 1:**
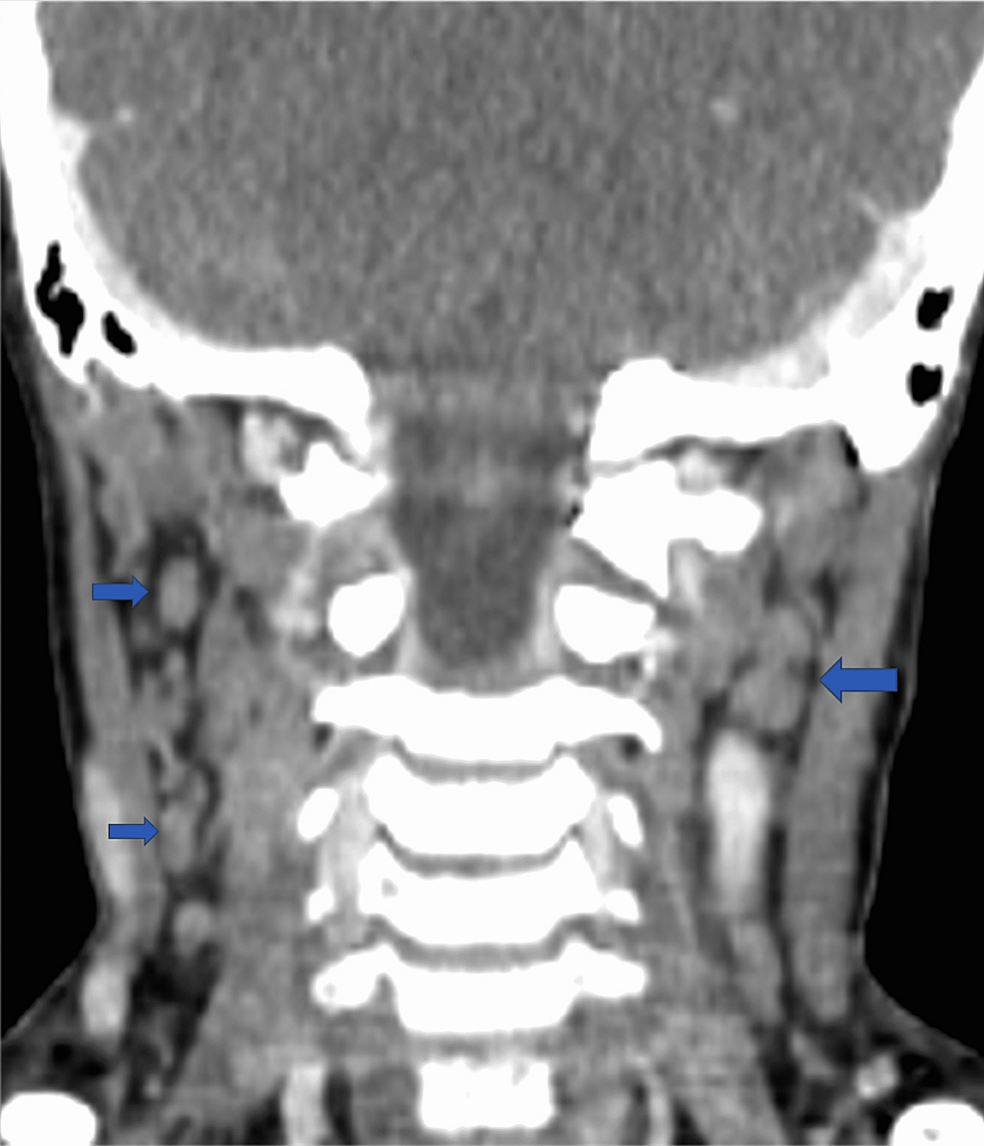
Sagittal CT scan of the neck showing multiple bilateral cervical lymphadenopathies (blue arrows).

**Figure 2:**
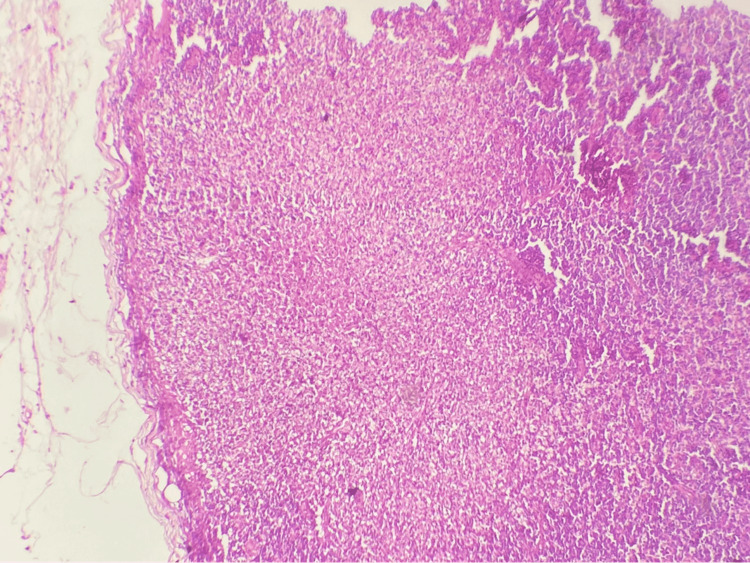
Low power view (hematoxylin and eosin x100) of the lymph node demonstrating extensive necrosis.

**Figure 3:**
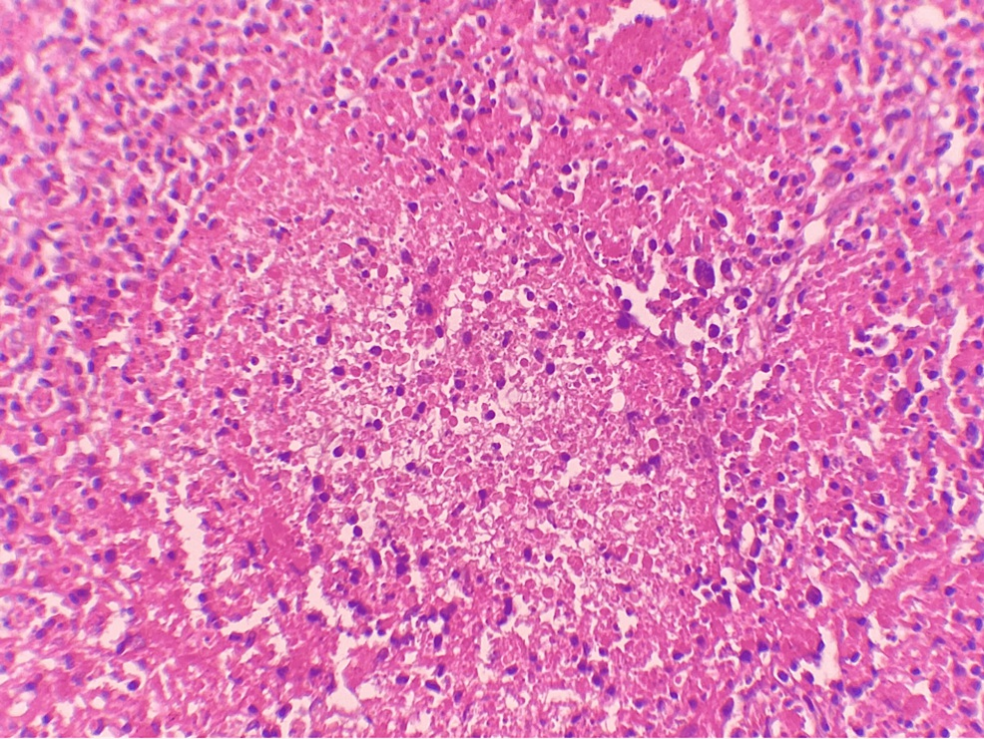
A high power view (hematoxylin and eosin x400) of the cervical lymph node biopsy showing an effaced architecture of the lymph node by necrotic foci without neutrophils.

An ultrasound evaluation of the neck showed multiple cervical lymphadenopathies, with the largest measuring 1.5 cm in diameter. In addition, the otolaryngological examination with laryngoscopy was normal. A computed tomography (CT) of the neck, chest, and abdomen revealed multiple cervical lymphadenopathies with the largest measuring 1.5 cm (Figure 1) without enlargement of lymph nodes in the axillary, mediastinum, or thoracic chains. Cervical lymphadenectomy with histological examination showed an effaced architecture with the association of large necrotic zones (Figure 2, Figure 3).

An immunohistochemical study demonstrates the abundance of CD68+ histiocytes confirming the diagnosis of KFD. The therapeutic approach was based on oral corticosteroid with prednisolone 60 mg daily for one week in front of persistent fever despite symptomatic treatment with paracetamol and non-steroidal anti-inflammatory drugs (NSAIDS). A favorable outcome was obtained after four weeks, characterized by a significant resolution of lymphadenopathy and systemic symptoms with a normalization of the laboratory values. No further recurrences or complications occurred to date after one year and a half following diagnosis.

## Discussion

KFD also known as histiocytic necrotizing lymphadenitis, was first described by Kikuchi and Fujimoto in Japan in 1972 [8,9]. It affects primarily lymph nodes of the cervical region. It’s a rare disease with sporadic cases described particularly in Asia, with 80% of cases reported in Japan [10], but more recently KFD has been reported all over the world [1]. KFD typically occurs in patients younger than the age of 40 with an average onset of 21 years and a female predominance [1] such as our patient. However, KFD can affect other age groups, including children. The clinical presentation of KFD is acute to subacute, mainly with cervical lymphadenopathy in more than 80% of cases, usually involving the spinal or jugular lymph nodes, as found in our patient. However, all lymph nodes regions can be involved or generalized [11,12]. Lymphadenopathies are firm, with a diameter of 0.5 to 3 cm, in some cases reaching 5 to 6 cm, movable, sometimes painful, and never suppurative [11]. In the literature, deep sites of lymphadenopathy are reported, such as mediastinal lymphadenopathy, intra-abdominal lymphadenopathy, hepatomegaly, and splenomegaly [1,13]. Cervical lymph nodes have no specific imaging features. Ultrasound and CT reveal non-necrotic oval nodes, commonly measuring between 1 and 3 cm [1]. As in the patient in the present case, multiple bilateral cervical lymphadenopathies did not exceed 1.5 cm in greatest diameter.

No laboratory study is pathognomonic for this pathology. Laboratory abnormalities associated with KFD include mainly an inflammatory syndrome with moderate granulocytopenia observed in 25-58% of cases [1]. In the current case, there was revealed leukopenia with mild neutropenia, lymphopenia, and anemia associated with an inflammatory syndrome. Serodiagnostic tests for HIV, toxoplasmosis, rubella, syphilis, and infectious mononucleosis are often negative.

The diagnosis is confirmed based on histopathological examination of the lymph nodes biopsy and immunohistochemical testing. It shows typically on microscopic examination as a partially preserved architecture with follicular hyperplasia. Three forms have been described in the literature: necrotic form (more than 50% of cases), proliferative form (30%), and xanthogranulomatous form (<20%) [14]. Histological examination, especially seen in sub-Saharan Africa, also helps to exclude the main differential diagnoses such as infectious lymphadenitis (particularly tuberculosis) and lymphomas. Immunohistochemical analysis reveals CD3+ cells as the majority of the cells present in the pathological areas of the lymph node. The cell population is predominantly composed of CD68+ histiocytes, while the lymphocyte population represents 20-50% of the total cell population, mainly CD8+ cytotoxic T-lymphocytes [14]. These heterogeneous results may be a reflection of the different immunological staging of KFD [14].

KFD continues to be an enigmatic disease, its etiology and pathogenesis are still unclear and there are no definite diagnostic criteria. The two most common hypotheses discussed in the literature are infectious and autoimmune conditions that may manifest similarly. Several infectious agents were supposed to incite KFD including EBV, HIV, human herpesvirus 6 (HHV-6), human T lymphotropic virus type 1 (HTLV1), parvovirus B19, and species of *Toxoplasma*, *Bartonella*, and *Brucella* [10]. However, there is no evidence that infection may directly cause KFD and several studies failed to detect these infectious agents in the involved lymph nodes [10,15]. The other suggested theory is that KFD may be related to an autoimmune disorder. Many case reports and studies have reported the association between KFD and systemic lupus erythematosus (SLE). KFD can occur before SLE or simultaneously [16]. In our case, a complete clinical workup was performed to eliminate infectious and autoimmune diseases, and histological analysis confirmed the absence of lymphoproliferative disease.

In the last years, cases of KFD have been described after infection with COVID-19 and have linked KFD with COVID-19 infection [3,4]. However, it is very uncommon for KFD to occur after vaccination, and only rare reports in the literature have described its occurrence mainly after influenza vaccination [17], human papillomavirus vaccination, and Japanese encephalitis virus vaccination [18]. More recently, four cases of KFD have been described after vaccination against COVID-19, and the main features of these cases are given in Table 1.

**Table 1:** Main features of cases of KFD post COVID-19 vaccination KFD: Kikuchi-Fujimoto disease; COVID-19: coronavirus disease 2019; mRNA: messenger ribonucleic acid

Author	Gender	Age	The interval between the first symptom or lymphadenopathy and vaccination	Vaccine administrated	Sites of lymphadenopathy
Soub et al. [5]	Male	18	10 days	mRNA vaccine	Supraclavicular, cervical and axillary
Tan et al. [7]	Male	34	17 days	mRNA vaccine	Axillary
Tan et al. [7]	Female	18	35 days	mRNA vaccine	Supraclavicular, subpectoral and axillary
Guan et al. [6]	Male	36	15 days	Inactivated COVID-19 vaccine	Cervical

All the patients were young and the most common sites of lymphadenopathy were the supraclavicular, cervical, and axillary nodes. All patients had a fever and other systemic manifestations as in our patient. The clinical course of these cases was favorable with symptomatic treatment to control fever; however, our patient didn’t improve with symptomatic treatment. We also noticed that cases reported with KFD post-COVID19 vaccination were mostly vaccinated with mRNA vaccine. Nevertheless, more data is required to determine whether KFD is specific to mRNA-based vaccines or can also occur in patients receiving other vaccine types.

In a study by Tan et al., the authors described the main features of previously published cases of lymphadenopathy following vaccination against COVID-19 [7]. The sites most involved were the axillary, supraclavicular and cervical lymph nodes ipsilateral to the vaccination site with reactive follicular hyperplasia or reactive lymphadenopathy on cytologic or histologic findings, and mainly there were reported after mRNA-based vaccines. These patients were either asymptomatic or had experienced only painless hypertrophy of the lymph node(s). In contrast, patients with KFD after the COVID-19 vaccination had both fever and other systemic symptoms. In addition, reactive lymphadenopathy after COVID-19 vaccination can appear as early as the first day after vaccine administration, but KFD tends to occur later. In the current case, as well as the other cases reported in the literature, lymphadenopathy appeared after several days ranging from 10 to 35 days. It is unclear how the vaccination could lead to the occurrence of KFD, but it could be potentially secondary to the viral or other antigens in the vaccine that may cause an aberrant immune response with some form of exaggerated T-cell mediated immune in vaccinated individuals [5,6]. 

Typically, in most cases, KFD has a favorable long-term prognosis with spontaneous resolution over an average of three months with regression of lymphadenopathy and resolution of systemic signs [1]. However, rare cases of disease relapse have been reported and can occur in 3-4% of KFD cases [19]. Nevertheless, due to an associated increased risk of developing SLE, close monitoring is recommended. Deaths are described in the literature concerning forms associated with systemic diseases, and corticosteroid therapy is recommended for reference treatment in such cases [20].

In summary, we report a rare case of a patient with KFD after vaccination against COVID-19. While the exact pathogenesis of the occurrence of KFD after vaccination is still unknown, it should be added to the list of potential factors associated with the occurrence of the disease.

## Conclusions

KFD is a rare benign disease that follows a self-limiting course and its pathogenesis remains still not fully understood. Its knowledge is interesting, which may allow limiting complementary examinations, especially invasive ones. Cervical lymphadenopathy differential diagnosis is diverse and the non-specific accompanying symptoms make the diagnosis difficult leading to misdiagnosis. Through our case, we illustrate the possible occurrence of KFD after the COVID-19 vaccination. Clinicians should be aware of this potential factor associated with KFD.
